# Sustaining agency after a fall: navigating healthcare and adjusting everyday life – a qualitative interview study

**DOI:** 10.1186/s12877-026-08418-2

**Published:** 2026-10-08

**Authors:** Sara Bergström, Kristina Rosengren, Catarina Wallengren, Hanna Falk Erhag, Ramona Schenell

**Affiliations:** 1 Institute of Health and Care Sciences, Sahlgrenska Academy, University of Gothenburg, Gothenburg, Sweden; 2 Region Västra Götaland, Department of Medicine, Geriatrics and Emergency Care, Sahlgrenska University Hospital, Mölndal, Sweden; 3 Centre for Person-centred Care (GPCC), Sahlgrenska Academy, University of Gothenburg, Gothenburg, Sweden; 4 Region Västra Götaland, Department of Acute Medicine and Geriatrics, Sahlgrenska University Hospital, Gothenburg, Sweden; 5 Administration of Eldercare and Welfare, The City of Gothenburg, Gothenburg, Sweden

**Keywords:** Falls, Healthcare services, Emergency department, Older adults, Qualitative content analysis, Relatives

## Abstract

**Background:**

Falls among older adults are a major public health concern and are associated with recurrent falls, functional decline, fear of falling, and reduced quality of life. Older adults presenting to emergency departments (EDs) following a fall are often discharged directly home despite ongoing rehabilitation and support needs. Although person-centred approaches are recommended in international guidelines, less is known about older adults’ and relatives’ experiences following an ED visit for a fall. This study aimed to describe older adults’ and their relatives’ experiences of a fall, including the ED visit and the transition back home.

**Method:**

A qualitative study design was used, involving semi-structured interviews with older adults aged 75 years and older and their relatives. Participants had attended the ED following a fall but were discharged without hospital admission. Seventeen participants (11 older adults and 6 relatives) were recruited. Data were analysed using qualitative content analysis according to Graneheim and Lundman.

**Results:**

The analysis generated one overarching theme, *Sustaining agency through recovery across care and everyday life*, which reflected how older adults and their relatives sought to manage daily life following a fall. Both groups described addressing immediate care needs while simultaneously adapting to longer-term changes in everyday life. Participants described difficulties navigating healthcare services to access follow-up care and rehabilitation, contributing to feelings of insecurity, financial strain, and increased responsibility. A persistent fear of falling affected daily activities and required adaptations and new roles for both older adults and their relatives. Despite these challenges, many participants demonstrated resilience and an ability to manage daily life following injury. Individual coping strategies and support from relatives were described as central to managing everyday life and healthcare contacts.

**Conclusions:**

Older adults discharged home after a fall may benefit from coordinated, person-centred follow- up that supports rehabilitation, recovery, and fall prevention. Despite variation in support needs, participants described a common need for clearer guidance and improved support structures, while also demonstrating considerable resilience and capacity for self-management.

**Clinical trial number:**

Not applicable.

**Supplementary Information:**

The online version contains supplementary material available at https://doi.org/10.1186/s12877-026-08418-2.

## Background

 Falls among older adults constitute a major public health concern worldwide [1], and are a leading cause of injury, hospitalization, reduced functional ability, and mortality [2, 3]. A substantial proportion of individuals aged 65 years and older experience at least one fall each year, and the incidence increases with advancing age [4]. In Sweden, falls are the type of accident associated with the highest number of deaths, hospital admissions, and emergency department (ED) visits [5]. Falls among older adults generate substantial societal costs, including healthcare, rehabilitation, and social services. Beyond these direct costs, informal care provided by relatives represents an additional burden [6, 7]. The consequences of falls extend beyond physical injury, as they are associated with reduced quality of life and loss of independence [8]. Older adults who seek emergency care following a fall are also at high risk of experiencing recurrent falls [9, 10] and often live with persistent functional impairment and fear of falling [11]. Many older adults who seek care after a fall already have impaired self-care capacity [12, 13], and approximately one-third of those discharged continue to experience fall-related disability three months later [14]. Fear of falling is common after a fall and may persist even in the absence of physical injury. This fear can lead to self-imposed restrictions on mobility and social participation, which may subsequently contribute to physical deconditioning and increased social isolation [15–17].

ED-initiated interventions, including patient education, structured referrals, and follow up, have been shown to reduce subsequent falls related visits. These findings suggest that, despite operational challenges, implementing fall prevention strategies in the ED is both feasible and effective [18]. These findings also highlights the importance of fall-risk assessment and multifactorial interventions in the ED, together with timely post-discharge follow-up for older adults at risk of recurrent falls [18–20].

There is growing recognition of the complexity inherent in fall prevention, as action-oriented interventions have not always demonstrated the expected effectiveness. This has been suggested to be partly due to a lack of individualized approaches [21]. Both the risk of falling and fear of falling are multidimensional phenomena influenced by medical history as well as social and psychological factors [22]. World guidelines for fall prevention and management, launched in 2022, recommend multifactorial fall prevention delivered through a person-centred approach [23]. Person-centred care (PCC) in the ED emphasizes partnership with patients and their families, engaging them in decision-making, and addressing psychosocial and cultural aspects, not just the medical condition [24]. However, PCC can be challenging to implement in the ED where fast-paced, high-pressure environments and time constraints often limit opportunities for detailed discussion and patient interaction [25].

PCC emphasizes a shift from purely biomedical thinking towards a greater focus on the individual [26]. Its ethical foundation requires that patients are recognized as unique individuals and active co–creators of their own care [27]. Within the international guidelines, a person-centred approach promotes shared decision-making and ensure that fall- risk assessments and prevention strategies are guided by the individual´s needs, preferences, and experiences, while incorporating the perspectives of older adults and caregivers [23]. Understanding how older adults and their relatives experience emergency care and the transition from the ED to home following a fall may provide important insights for the development of person-centred fall prevention strategies and post-discharge support.

Recovery after a fall often requires support from both healthcare professionals and informal caregivers, but dependence on relatives may contribute to caregiver burden, guilt, and anxiety [28]. Involvement of relatives can create complex dependency dynamics, as relatives strive to support autonomy despite limited resources and insufficient guidance from healthcare professionals [29]. A recent qualitative evidence synthesis exploring the perspectives and experiences of community-dwelling older adults who have experienced falls [30] highlights a range of strategies that may help healthcare providers develop person-centred care plans that motivate individuals, to take preventive action against future falls. However, the authors also state that further research across a wider range of contexts is needed, as the structure of different health and social care systems may shape these experiences and related factors.

Despite the high incidence of fall-related injuries, Sweden lacks national guidelines for fall prevention [31], and research indicates that older adults presenting to EDs following a fall are frequently discharged directly home without structured follow-up or coordinated preventive interventions [32, 33]. Previous research examining older adults’ experiences following ED visits more broadly has identified challenges in continuity of care, including gaps in information, coordination, and follow-up [34]. However, these findings are not specific to older adults seeking emergency care following a fall.

Although previous research has explored older adults’ experiences of falls and fall prevention, less is known about the period following a fall-related ED visit among those discharged directly home without hospital admission. In particular, greater understanding is needed of how older adults navigate healthcare and follow-up while managing their recovery and changes in everyday life, as well as how relatives experience and contribute to this process. Exploring these perspectives may help identify support needs that extend beyond the treatment of the immediate fall-related injury and inform person-centred follow-up after ED discharge.

Therefore, this study aimed to describe older adults’ and their relatives’ experiences of a fall, including the fall itself, the ED visit, and the transition back home.

## Method

### Study design

This qualitative study employed individual semi-structured interviews with older adults and their relatives and was reported in accordance with the Consolidated Criteria for Reporting Qualitative Research (COREQ) guidelines [35]. The study was situated within an interpretative qualitative methodological approach, consistent with qualitative content analysis as described by Graneheim and colleagues [36–38], acknowledging that qualitative analysis involves varying levels of abstraction and interpretation while remaining grounded in participants’ accounts. An inductive approach was applied to allow the analysis to be guided by the data in relation to the study aim rather than by a predefined theoretical framework. Qualitative content analysis was considered appropriate as it enabled a systematic exploration of similarities and differences in participants’ experiences while allowing both descriptive content and underlying meanings across the data to be identified and interpreted.

### Study setting

This study was conducted at one of Sweden’s largest university hospitals, which provides care to more than 226,000 patients annually across its six EDs. The study setting was one of these EDs, serving patients aged 16 years and older and managing primarily orthopaedic and medical conditions.

### Recruitment and participants

Community–dwelling older adults aged ≥ 75 years and their relatives were eligible for inclusion if they had experienced a fall and received care in the ED without subsequent hospital admission. Those living in residential care facilities, who did not speak Swedish, or who were unable to understand information or provide informed consent were excluded. The severity of injuries sustained following the fall varied considerably, ranging from no fracture or minor fractures requiring no further follow - up to humeral and wrist fractures requiring surgical treatment. Emergency staff (registered nurses and nursing assistants) identified potential participants during a two-week period in November 2024 and provided them with written information about the study to take home. The first author subsequently contacted potential participants by telephone approximately two to three weeks after the ED visit. Those who agreed to participate were asked whether they had a relative whom the researcher could contact. The relatives were first sent written information about the study and were subsequently contacted by telephone by the first author and invited to participate. In this context, the term “relatives” referred to a person identified as significant by the participant who did not necessarily have to be a family member. The interviews took place at a location chosen by the participants, either in their homes or in a private room at the hospital. All interviews were audio- recorded and transcribed verbatim by the first author.

Twenty-six older adults were invited to participate, of whom eleven agreed. Eight relatives of these participants were contacted and six agreed to participate (three daughters and three spouses). In total, seventeen participants were included in the study, the majority of whom were women. Table 1 presents participant characteristics. Following completion of the interviews, the research team assessed the adequacy of the data in relation to the study aim. The data were considered sufficiently rich and varied, capturing a range of experiences relevant to the phenomenon under study and providing an adequate basis for identifying patterns and developing meaningful categories in the qualitative content analysis. If the data had been considered insufficient in terms of richness, depth, or variation, an additional round of recruitment and interviews would have been conducted. The decision not to recruit additional participants was therefore based on the adequacy and quality of the data in relation to the study aim and the analytic process.

**Table 1:** Participant characteristics

Participants, *n*	Older adults	Relatives
	11	6
Women, n	9	4
Men, n	2	2
Mean age, years	82.5	66
Age range, years	75–89	47–89
**Educational level**,** n**		
Primary school / Elementary school	1	1
Upper secondary school	3	0
University / College / Higher education	7	5

### Data collection

The interviews were conducted individually using a semi-structured interview guide with open-ended questions, focusing on the participants’ experiences of the fall incident and the subsequent care, with particular emphasis on the personal impact of the incident. The interview guide was developed collaboratively by all authors in accordance with the study aim and was informed by Kvale and Brinkmann [39]. Two separate but closely related interview guides, one for older adults and one for relatives, were developed and are presented in Supplementary File 1. Interviews lasted between 18 and 62 min, with a mean duration of 37 min. Interview duration varied according to participants’ individual experiences and the extent to which they elaborated on these during the interviews.

### Analysis

An inductive qualitative content analysis, in accordance with Graneheim and Lundman [36–38], was conducted. Initially, the authors most closely involved in the analysis (SB, RS, KR) read all interviews repeatedly to gain a comprehensive understanding of the data. They then independently identified, condensed, and coded meaning units from a selection of interviews. The coding was compared to explore similarities and differences in interpretation. Data segments relevant to the study aim were identified and verified. SB subsequently assumed primary responsibility for coding the remaining material and developing the manifest category structure. Microsoft Word was used during the initial coding process, while Microsoft Excel was used to organise and manage the codes and support the subsequent analytical process. Codes were compared based on similarities and differences and organised into categories reflecting the manifest content of the data. At the latent level, subthemes and themes were developed through interpretation of the underlying meanings across categories. 

Examples illustrating the analytical process from meaning units and condensed meaning units to codes, subthemes and themes are presented in Table 2. 

### Trustworthiness

Trustworthiness was considered in terms of credibility, dependability, and transferability throughout the research process. Credibility was supported by the systematic analytical process and the involvement of several researchers in the analysis. Three authors (SB, RS, KR) initially analysed a selection of interviews independently, and similarities and differences in coding and interpretation were subsequently discussed. Categories, subthemes, and themes were repeatedly discussed and refined within the research group, allowing alternative interpretations to be considered. Representative quotations are provided in the findings to illustrate the participants’ perspectives and demonstrate the grounding of the interpretations in the data.

Dependability was supported by maintaining a structured analytical process and by continuously moving between the interview text, meaning units, codes, categories, and emerging interpretations. Analytical decisions and interpretations were discussed within the research group throughout the process.

All researchers were registered nurses with professional backgrounds in healthcare, and four (RS, KR, CW, HFE) held PhDs in Health and Care Sciences. SB was clinically active in an emergency department caring for older adults with fall-related injuries and therefore had particular pre-understanding of the study context. The researchers critically reflected on and discussed how their professional experiences and pre-understandings could influence the analytical process. The involvement of researchers with different clinical and research experiences enabled different perspectives on the data to be considered during the interpretative process.

To facilitate assessment of transferability, the study context, participant characteristics, recruitment and data collection procedures are described, and representative quotations are provided to allow readers to assess the applicability of the findings to other contexts.

**Table 2:** Example of meaning unit, condensed meaning unit, code, subtheme, and theme

Meaning Unit	Condensed Meaning Unit	Code	Subtheme	Theme
And there were quite a few of us involved. There was me, and there was the neighbour, who was also there and went in to see Mum every day. *(Relative 15)*	There were several of us involved. The neighbour also went to see Mum every day.	Support from several people	Relying on support in care encounters	Navigating health and care services
Actually, I feel… I feel that I need at least some help with grocery shopping or cleaning at home, because I can’t manage on my own. So, I do need help, but on the other hand, I only have a small pension… *(Older adult 10)*	Feels a need for some help with shopping or cleaning at home, as unable to manage alone, but has limited pension.	Wants help but has a low pension	Relying on support in care encounters	Navigating health and care services
Thought it was just something that happens to old people, then realized I’m 85 myself. (*Older adult 6)*	“Thought it happens to old people, then realized I’m 85 myself.”	Just something that happens to old people.	Adapting to a new role	Adjusting to a new life situation
Yes, I do think it’s a rapid recovery, it really is. But it wouldn’t have been possible without consistent training. It’s the persistent effort, and largely because I want to be a good enough dog owner to XXX. He needs to run and expend his energy. (*older adult 7)*	It’s been a rapid recovery, but only possible through persistent training, driven by a desire to be a good enough dog owner to XXX, who needs to run and expend energy.	Exercising to regain recovery and be a good dog owner.	Maintaining autonomy through self-management	Adjusting to a new life situation
Now, I’m a bit afraid of it, to be honest. Before this happened, I had fallen several times when I was out with my other knee, so I think I’ve become more cautious about going out at all. *(Older adult 8)*	Fear after repeated falls has made me more cautious about going out.	Repeated falls leading to avoidance of going out	Managing perceived fall risk	Adjusting to a new life situation

### Ethics

This study was approved by the Swedish Ethical Review Authority (2022-03409-01) and conducted in accordance with the principles of the Declaration of Helsinki [40]. The emergency department staff (registered nurses and nursing assistants) who identified potential participants had no prior relationship with the participants and provided them with written information about the study to take home. Subsequently, a researcher contacted potential participants by telephone and provided additional oral information about the study. Participants were informed that participation was voluntary, that they could withdraw from the study at any time without providing a reason, and that confidentiality would be maintained. Written informed consent was obtained from all participants.

## Results

The overarching theme, *“Sustaining agency through recovery across care and everyday life”*, reflects how older adults and their relatives sought to manage daily life following a fall. Both groups described addressing immediate care needs while simultaneously adapting to longer-term changes in everyday life, often characterized by restraint in help-seeking and a strong sense of persistence. The findings show that this striving to manage was shaped by two interrelated processes: *“Navigating health and care services”* and *“Adjusting to a new life situation.”* Across these processes, older adults and relatives described experiences of decision-making along care pathways, varying access to support, and a substantial responsibility for coordinating care and rehabilitation. Participants also described how everyday life was reshaped in response to perceived fall risk, changing roles, and the need for ongoing self-management. These efforts were directed toward maintaining independence and making everyday life manageable despite pain, functional limitations, and uncertainty.

An overview of the findings is presented in Table 3.

**Table 3:** Overview of main theme, themes, and subthemes

Main theme	Theme	Subtheme
Sustaining agency through recovery across care and everyday life	Navigating health and care services	Making health care decisions Relying on support in care encounters Assuming personal responsibility for follow-up and rehabilitation
	Adjusting to a new life situation	Managing perceived fall risk Adapting to a new role Maintaining autonomy through self-management

### Navigating health and care services

The theme *“Navigating health and care services”* reflects the challenges associated with seeking care and coordinating follow-up across multiple healthcare providers and levels of care. Both older adults and their relatives described uncertainty arising from limited information about available services and support which they were entitled. This uncertainty influenced decision-making and contributed to participants assuming responsibility for accessing and coordinating care. Older adults described having to make decisions about where and when to seek care, often relying on practical and emotional support from relatives. They also reported carrying substantial responsibility for coordinating follow-up care and rehabilitation. In some cases, financial constraints further influenced their ability to access and accept community-based support services.

#### Making healthcare decisions

This subtheme captures older adults’ uncertainty and lack of preparedness when navigating different healthcare providers, sometimes in the context of financial strain. The period immediately following a fall involved a series of decisions, including whether the injury was serious enough to warrant seeking care and, if so, where to seek help. Older adults described weighing symptoms, consulting family members or neighbours, and considering practical circumstances such as the time of day and the availability of services.

Several older adults found it difficult to determine the most appropriate level of care and described being referred between primary care, radiology services, and emergency departments, sometimes without a clear understanding of where responsibility for care rested. Previous negative experiences and recent encounters with long waiting times contributed to reluctance to seek emergency care, even when symptoms persisted. As one older adult explained:

“*No*,* it wasn’t that dangerous*,* no*,* but I got up and saw that the wrist was crashed*,* yes*,* so I thought it would probably be best to go home… so I called one of my grandchildren because I knew she was home*,* that she could come and wrap the wrist*,* since it was the right hand*,* and then she said*,* ‘Shall we go to visit the health care center?’ But no*,* I said*,* what would they do there*,* so I decided to wait and see*,* and then we’ll see what to do. Later*,* my son called and said*,* ‘Shall we visit ED’ and yes*,* okay*,* we’ll do that” (Older adult 11)*.

While older adults generally considered the assessment and treatment provided in the ED to be appropriate, both older adults and their relatives described uncertainty regarding the transition from acute care to recovery at home. The rationale for aftercare decisions, the expected follow-up process, and responsibility for coordinating ongoing care and rehabilitation were frequently perceived as unclear, leaving participants uncertain about how recovery would be supported after discharge, what services might become available, and who was responsible for ensuring that follow-up care needs were addressed. Participants also described uncertainty about future support needs related to care, housing, and everyday functioning. This uncertainty was reinforced by an injury-focused approach that was perceived as overlooking the older person’s broader situation and longer-term support needs.

Despite these concerns, older adults expressed trust in healthcare professionals’ clinical judgement and prioritization of patients. Criticism was primarily directed towards the healthcare system as a whole and was often framed in terms of limited resources rather than dissatisfaction with individual healthcare professionals. One older adult reflected:

*“Well*,* I understood that it was a lot (going on) during the last two hours of the waiting time*,* I actually had to lie down*,* and then I noticed that ambulances were coming and such things*,* and during those two hours*,* two emergency alarm occurred. So I understand that there were others who had to be prioritized*,* so I wasn’t surprised.” (Older adult 13)*.

#### Relying on support in care encounters

This subtheme captures how information sharing, continuity, and involvement of relatives facilitated care and recovery. Older adults described needing assistance throughout the care trajectory, from the immediate aftermath of the fall and the ED visit to aftercare and recovery at home. Family members and friends were often described as crucial sources of both emotional and practical support, accompanying older adults to healthcare appointments, asking questions on their behalf, and helping them recall and interpret information afterward.

Feeling well cared for in the ED was associated with being acknowledged and seen, receiving clear and comprehensible information, and being offered basic comforts such as a place to rest, food, or drinks. These experiences contributed to a sense of trust and safety. In contrast, older adults described how a lack of support amplified feelings of insecurity. This included prolonged waiting while in pain, uncertainty about whether they would be seen by a physician, and uncomfortable waiting conditions, such as sitting for extended periods on hard chairs despite pain and fatigue. Information was sometimes communicated quickly, leaving little time to process it or ask questions.

Many older adults described that questions regarding casts, orthoses, pain management, and what to expect after discharge emerged only later, once they had returned home. At that point, follow-up pathways and points of contact were often perceived as unclear or difficult to access. In these situations, relatives frequently acted as a practical bridge between healthcare services and the older person, helping to clarify information, manage prescriptions, and arrange follow-up contacts, particularly when the older person felt overwhelmed, fatigued, or unsure what questions to ask during the healthcare encounter.

Relatives described their role during healthcare encounters as listening carefully and retaining information so that instructions could be repeated and clarified afterwards. However, they did not always feel that healthcare professionals actively acknowledged or involved them in this supportive role. One relative described this as follows:

“*Should I*,* I don’t really know*,* you don’t see the whole person in any way*,* and that part*,* they (healthcare professionals) didn’t care what happened when she gets home. With painkillers*,* if she (older person) hadn’t had any relatives then she wouldn’t have had anything at home then she get there*,* she couldn’t go and visit a pharmacy.” (Relative 15)*.

Beyond informational and practical support, access to assistance was also shaped by financial circumstances. For some older adults, the fall resulted in new or increased expenses related to transport, rehabilitation, and home care services, creating an additional financial burden. These costs influenced decisions regarding healthcare utilisation and the support considered feasible following the fall. Financial strain could contribute to delaying or avoiding healthcare contacts or declining home care services despite evident functional needs. Limited financial resources also affected whether support with shopping, cleaning, or other home care services was considered affordable. In some cases, relatives and friends stepped in to help navigate social services or provide practical and financial support.

*“Actually*,* I feel that I need some help*,* at least with shopping and cleaning at home*,* because I can’t manage everything on my own. So*,* I do need help*,* but at the same time*,* I have only a small pension.” (Older adult 10)*.

#### Assuming personal responsibility for follow-up and rehabilitation

Participants described an expectation that they would independently manage subsequent steps in the recovery process.

Relatives described taking responsibility by being present during healthcare encounters, arranging transport, collecting medications, assisting with home arrangements, and monitoring ongoing needs. This responsibility was often portrayed as a natural part of the family role, even when it had consequences for work or family life. Relatives also highlighted that older spouses could face challenges in taking on this role because of their own illness, disability, or limited resources. Nevertheless, they often remained involved in the background, for example by ensuring that the older person sought care and had the practical means to maintain contact with healthcare services.

Both older adults and relatives described responsibility as actively ensuring that referrals, follow-up appointments, and rehabilitation services were initiated and maintained. They reported having to make contacts themselves and coordinate between healthcare providers, rehabilitation services, and primary care. Even among older adults who felt capable of managing these tasks, there was a concern that people with less energy, poorer health, or limited support might struggle to navigate the system. Participants described occasions when planned follow-up did not occur as expected, leaving them responsible for arranging physiotherapy or occupational therapy on their own. As one older adult explained:

*“…When you’re done at the hospital*,* you’ll have to find a physiotherapist yourself… You’ll be a bit left behind…” (Older adult 1)*.

Despite these challenges, older adults described a strong commitment to their own rehabilitation. Taking responsibility was closely linked to a desire to recover and regain independence. When information was lacking, some sought answers independently and attempted to fill perceived gaps in knowledge. One older adult described searching for information online when explanations had not been provided during the healthcare encounter:

*“And then I read a bit about it on my phone*,* and that´s when I understood that it can take between six and eight weeks before it´s completely better” (Older adult 6)*.

Older adults also described setting their own rehabilitation goals and making personal judgments about when and how to progress in their recovery. However, responsibility became more difficult to manage when energy levels were low, symptoms persisted or attempts to contact rehabilitation services went unanswered. In these situations, participants described feeling caught between services and left to manage complex care needs on their own.

Even so, older adults emphasized that they took this responsibility seriously, and some expressed self-blame when rehabilitation did not proceed as expected or when complications arose. As one older adult reflected:

*“I carefully read the message about what it was regarding*,* and that I was recommended to come in*,* get in touch with our rehab services*,* and probably end up there. But I never got any response from them when I tried. And maybe that was my own fault*,* maybe I should have followed up more persistently. But I really wasn´t in the right state of mind or condition to fight for getting into rehab in that situation. No” (Older adult 8)*.

### Adjusting to a new life situation

The theme *“Adjusting to a new life situation”* describes how both older adults and their relatives experienced changes in everyday life following the fall. Participants described shifts in roles, altered routines, and heightened feelings of vulnerability, often related to concerns about recurrent falls and uncertainty regarding future health and functioning.

Within this broader process of adjustment, older adults described striving to return to a sense of normality while adapting to physical limitations, restrictions, and changes in daily activities. To maintain independence and continue managing everyday life, they adopted a range of practical and behavioural strategies aimed at preserving autonomy and functioning despite ongoing challenges.

#### Managing perceived fall risk

This subtheme captures how older adults and their relatives managed perceived risks of recurrent falls in everyday life. Following the fall, older adults described heightened awareness of their vulnerability and an ongoing concern about falling again. These concerns shaped how they navigated daily activities and led to various strategies aimed at reducing risk and maintaining safety.

Older adults described becoming more cautious when moving about outdoors, paying increased attention to environmental hazards, and adapting daily routines. Many reported constantly monitoring ground surfaces, weather conditions, and footwear, particularly during winter, as part of their efforts to prevent another fall. They also linked their perceived fall risk to functional impairments and health conditions, including balance problems, dizziness, osteoporosis, and osteoarthritis.

Managing perceived fall risk often involved balancing safety with participation in everyday activities. Older adults described modifying or avoiding certain activities, such as using public transport, going outdoors alone, or walking a dog. While these adaptations were intended to reduce risk, they could also restrict everyday mobility and participation.

Relatives similarly described taking an active role in managing perceived fall risk. They reported that some older adults underestimated their limitations and engaged in activities perceived as unsafe, whereas others restricted themselves excessively and needed encouragement to remain active. Relatives therefore sought to support a balance between caution and continued engagement in everyday life.

Relatives also described having a personal stake in preventing future falls. Some were dependent on the older person’s support in everyday life, while others experienced difficulties providing the level of assistance they wished because of their own health problems, work responsibilities, or family commitments. Consequently, relatives often assumed an active preventive role by monitoring conditions that could increase fall risk and encouraging precautionary behaviours. This included calling or sending messages to remind older adults about slippery conditions, appropriate footwear, or the use of assistive devices. One relative described these efforts as follows:

*“She tended to be cautious*,* concerned that it might cause pain. On particularly icy days*,* I could call or text her in the morning to remind her to wear traction devices*,* as the ground was slippery” (Relative 16)*.

#### Adapting to a new role

This subtheme captures how older adults and their relatives adapted to changing roles and self-perceptions following the fall. Participants described how the fall challenged established identities, relationships, and expectations regarding independence, capability, and support.

Older adults described how the fall could alter the way they viewed themselves, shifting from being independent and self-reliant to needing help from others. This transition was often emotionally charged and associated with feelings of embarrassment, frustration, and vulnerability. For some, the fall symbolized aging and frailty in a way that was difficult to accept. Participants described irritation directed both at the event itself and at what it seemed to represent, namely becoming “old,” “fragile,” or dependent on others. One older adult described this experience as follows:

*“Luckily*,* only one person saw the fall when it occurred. Maybe it doesn’t matter*,* but to me it does. They think that old ladies trip over all the time*,* and maybe they do too” (Older adult 12)*.

Relatives likewise described having to adjust how they perceived the older person and their own role in relation to them. For some, it was difficult to reconcile the new situation with a long-established image of a parent as capable, independent, and strong. Changes in responsibility and expectations required ongoing negotiation within families regarding support needs, decision-making, and the acceptance of help at home. Relatives also described feelings of guilt when they were unable to provide as much support as they wished, as well as tensions between siblings when responsibilities were shared unequally or opportunities to contribute differed.

Both older adults and relatives described adapting to new roles as an ongoing process of renegotiating expectations about needs, capabilities, and dependence. One relative reflected:

*“But when you´ve spent a whole life with your mother handling everything*,* always being the strong one*,* she continues to be that way. The idea that mom could become weak and not manage things herself was a shift my brain couldn´t process quickly enough*,* I have to say” (Relative 15)*.

In adapting to these changing roles and circumstances, participants described a range of coping strategies. For some older adults, adaptation involved accepting the fall as a life event and reframing it as an opportunity to learn or to recognize the care and support available from others. Some described becoming more aware of the kindness of family, friends, and healthcare professionals, and feeling less isolated than before the fall. For others, coping was characterized by frustration, self-blame, and repeated reflections on what could have been done differently, even while acknowledging that the event could not be changed.

Older adults also described adapting through practical adjustments in everyday life. This included reducing activities, postponing plans, and temporarily putting aspects of life on hold when multiple challenges accumulated. At the same time, they continued to express a strong desire to return to normality and regain their previous level of independence.

#### Maintaining autonomy through self-management

This subtheme captures how older adults sought to maintain autonomy through self-management, problem-solving, and adaptation in everyday life following the fall. Despite physical limitations and ongoing recovery needs, older adults described themselves as capable of managing daily life by finding new ways to accomplish necessary tasks. Capability was perceived as involving more than physical function alone; it also encompassed initiative, determination, and a willingness to make things work under changed circumstances.

Older adults emphasized the importance of remaining active participants in their own lives rather than becoming passive recipients of care. Maintaining autonomy involved taking responsibility for oneself, directing the assistance received from others, and ensuring that everyday tasks continued to be accomplished, even if they had to be performed differently than before the fall.

Putting this autonomy into practice during the early recovery period often required creativity and persistence. Older adults described having to relearn and adapt everyday routines such as personal hygiene, dressing, cooking, and household tasks in response to pain, reduced mobility, or temporary loss of function. They developed practical solutions and compensatory strategies, sometimes using unconventional methods, to maintain self-care and everyday functioning. Managing personal hygiene was a recurring example, with participants describing a process of trial and error through which they gradually learned what was possible and how activities could be performed safely. Self-management also extended to rehabilitation and fall prevention, including strength training, the use of mobility aids, and modifications to the home environment such as removing rugs and other potential hazards.

Relatives described their own role as balancing concern for the older person’s safety with respect for their autonomy. While they provided practical assistance and encouraged precautionary behaviours, they also sought to avoid undermining independence or conveying a sense of incapability. This balancing act could be challenging, particularly when they felt a need to intervene more actively than the older person wished. One relative described this tension as follows:

*“Yes*,* I think I may have been a bit too pushy at times and*,* in some situations*,* almost treated her as if she were incapable of making her own decisions. I was trying to help*,* but she saw it as me constantly pointing out her age. I’m very aware of that*,* which is why I think it’s important that she is allowed to do the things she can still manage on her own.” (Relative 17)*.

Both older adults and relatives described preserving independence as the overarching goal, even when temporary assistance was required. Participants described support from neighbours, family members, and friends as enabling autonomy rather than replacing it. In this way, informal support created conditions for continued independence and everyday functioning, while reducing reliance on formal care services.

## Discussion

Both older adults and relatives emphasized that preserving independence remained the overarching goal, even when temporary assistance was required. Participants described support from neighbours, family members, and friends as enabling autonomy rather than replacing it. In this way, informal support created conditions for continued independence and everyday functioning, while reducing reliance on formal care services.

This study described the experiences of older adults and their relatives following a fall that required an ED visit but not hospitalization. The findings illustrated how individuals who had experienced a fall, together with their relatives, faced challenges in accessing healthcare, managing daily life, and coping with the fear of falling, with individual circumstances influencing these experiences. Healthcare services often functioned adequately in treating fall–related injuries but failed to provide sufficient support during rehabilitation. The participants expressed that they were left on their own to determine both appropriate follow-up care and who was responsible for providing it. Despite challenges, older adults generally managed on their own, demonstrating adaptability and problem-solving, often with support from relatives and friends. Healthcare was generally perceived as adequate for medical issues but offers limited holistic follow-up for daily support, pain management, and rehabilitation. Nevertheless, the participants demonstrated considerable resourcefulness, taking responsibility for their health and showing initiative in managing their recovery. Previous studies have highlighted reduced self-care capacity among older adults attending the ED after a fall [11, 13]. However, this study provides a more nuanced understanding of the strengths and capabilities present in this population. The same applies to relatives who were actively involved and provided support both in managing daily life and in helping older adults regain confidence to resume activities.

Several participants expressed uncertainty about where to seek care and hesitated to go directly to the ED, even in cases where injuries were severe, such as a dislocated wrist or an inability to walk. This reluctance to seek emergency care was partly related to negative perceptions and previous experiences of the ED, which was described as an uncomfortable environment characterised by limited communication. Participants who expressed satisfaction with the care they received highlighted the compassionate treatment in the ED, including clear information, respectful interactions, and attention to basic comfort needs. Previous research has identified long waiting times, unpleasant waiting conditions, and insufficient communication as key challenges in older adults’ experiences of emergency care [41]. Participants reported limited care options, as they were directed to the ED by primary care providers, both immediately after the fall and for subsequent complications. These findings suggest that difficulties in navigating the healthcare system may contribute to delayed care-seeking and uncertainty following a fall. The challenge of identifying the appropriate level of care and establishing optimal care pathways for older adults is well recognized. Decision-making regarding the appropriate level of care is complex, with multiple individual and structural factors influence help-seeking behaviour. In many cases, the ED remains the most appropriate setting despite initial hesitation to attend [42–44] while evidence also supports the need for a more holistic perspective to the care of older adults in emergency settings [45].

There was also an economic dimension to seeking care, accessing social services, and receiving rehabilitation, which led some to avoid healthcare visits, assistance, and treatment due to financial concerns. These findings underscore the importance of recognizing each older adult´s unique circumstances when planning and delivering person-centred care. Individuals with lower socioeconomic status have been shown to have at higher risk of falls, highlighting the importance of considering social and economic inequalities in fall preventive strategies [46]. Previous research acknowledge that family support and economic resources influence older adults´ perspectives on falls, but provide limited insight into these relationships, highlighting the need for further research in this area [30]. Alongside financial concerns, fear of falling emerged as another important factor shaping participants’ everyday experiences following a fall. Fear of falling appeared to influence older adults’ gait patterns and contributed to activity restriction. This fear was not limited to the individuals themselves but also affected their relatives, who, for example, reported calling to warn about slippery conditions. Fear of falling following a fall has been widely reported in previous research [47] and has been identified as an independent predictor of subsequent falls [48]. However, fear of falling is a complex phenomenon that may reflect both adaptive and maladaptive processes [49]. Therefore, when interpreting these findings, it is important to consider whether the observed responses reflect protective caution or harmful avoidance. From a clinical perspective, regardless of whether concerns are adaptive or maladaptive, fear of falling warrants clinical attention and management [8]. Addressing fear of falling requires an individualized and person-centred approach, as the meaning and consequences of fear vary between individuals. Healthcare providers play a central role in motivating older adults to recognize their own risk and understand that future falls may be preventable [50]. In terms of fall prevention, our findings highlight the importance of understanding how preventive interventions can be initiated, as well as the need to take advantage of the “teachable moment” that may arise following a fall-related injury to prevent subsequent falls and support rehabilitation. Research suggests that older adults are often more receptive to fall prevention interventions following a fall event [51].

The role of relatives in fall management and fall prevention has previously been highlighted as crucial and may represent a turning point in family dynamics [52, 53]. Relatives play a key role in preventive efforts, and taking the context of the older person´s family relationships into account may enhance fall prevention interventions. Such interventions should also address the health and wellbeing of caregivers [52, 53]. In our study, we observed that relatives’ fear of future falls was closely linked to their own wellbeing and their ability to cope with the possibility of another fall. Therefore, fall prevention should combine physical rehabilitation with emotional support and approaches that promote independence and self-determination among both older adults and their relatives. Individualized approaches should also incorporate the perspective of informal caregivers by assessing caregiver burden, offering guidance and emotional support, and encouraging safe independence rather than overprotective practices [54]. Turning to another key finding, older adults assumed considerable responsibility for their own rehabilitation and, when they felt they had not done enough, often blamed themselves. These findings suggest that many participants lacked guidance from healthcare professionals and were often left with unanswered questions following the ED visit. These findings indicate a need for health professionals to provide clearer information regarding follow-up care, including treatment, rehabilitation, and relevant healthcare services following a fall. Such information should be tailored to the individual´s circumstances, resources, and preferences, in accordance with person-centred principles. These findings also suggest that engaged family members should be recognized and involved in information – sharing processes, as this may equip relatives with the knowledge and resources needed to support fall – prevention efforts.

Staff attitudes toward fall risk assessments and prevention indicate a need for greater knowledge and awareness regarding modifiable risk factors among older adults presenting after a fall [55]. However, responsibility for missed opportunities for fall prevention does not rest solely with healthcare professionals. The absence of standardized fall-risk screening, dedicated prevention services, and systematic rehabilitation may also contribute to recurrent falls [23, 56, 57]. Fall prevention programmes typically involve multiple points of contact across different professional groups and healthcare providers, including municipal services, primary care and hospitals. This places high demands on communication and a clear delineation of responsibilities [58] although such programmes have been shown feasible and effective [18].

Falls among older adults are often not isolated events but may represent an early sign of frailty. Research has shown that older persons presenting to the ED after a fall frequently have underlying frailty, multimorbidity and an increased risk of functional decline, recurrent falls, hospitalization, and mortality [59]. Our study illustrates a range of challenges faced by older adults with fall - related injuries, while also highlighting the diverse resources and capacities they draw upon to manage their situations. A common denominator, however, was perceived lack of information in the ED and support following discharge. These findings highlight the need for person-centred care that builds on the individual´s resources while identifying and addressing their support needs. Taken together, our findings, in combination with current knowledge regarding fall -related risks and the importance of preventative strategies, suggest that older adults who are discharged directly home following a fall may benefit from structured follow-up focusing on wellbeing, rehabilitation, and fall prevention, delivered through a person-centred approach.

### Strengths and limitations

There is a need for further research describing older adults’ and their relatives’ experiences following an ED visit after a fall, particularly in different healthcare contexts, to inform the development of follow – up strategies [30]. The findings of the present study provide important insights that may contribute to the development of both clinical practice and person - centred approaches. A strength of the study was the inclusion of both older adults and relatives, which enabled the exploration of experiences from multiple perspectives. Furthermore, qualitative interviews were suitable for capturing participants’ perspectives, while qualitative content analysis facilitated the systematic identification and interpretation of patterns within their experiences. The number of interviews and the variation among participants, including differences in age and the inclusion of both spouses and daughters, provided rich and varied experiential data. Furthermore, the composition of the research group, including both a nurse with experience from the ED and researchers with external perspectives, strengthened the credibility of the analysis. However, several limitations should be considered. The inclusion of only Swedish–speaking participants may have limited the range of perspectives and could affect the transferability of the findings. Individuals who agreed to participate may have had a greater interest in the topic than those who declined participation. In addition, older adults with a high disease burden and/or living with frailty may have had less energy or opportunity to participate in the study, which may have influenced the range of experiences represented. As participants were recruited from a single ED within one healthcare region in Sweden, the findings may not fully reflect experiences in other regions, healthcare systems, or national contexts. Furthermore, the organization of healthcare and follow-up services may differ between settings and countries, which could influence the transferability of the findings.

## Conclusion

This study supports the well – established need for coordinated care for older adults discharged home after a fall, particularly with regard to clear information about responsibility for follow- up, available and recommended support services and patients’ rights. There is considerable variation in support needs among both older adults and their relatives. The economic consequences of falls should also be considered when planning fall prevention interventions. Despite this variation, there is a shared need for improved structures to support safe rehabilitation and recovery. At the same time, older adults represent a resourceful group with substantial knowledge, resilience and adaptability. The findings emphasize the importance of person – centred interventions, with care tailored to individual needs, preferences and circumstances following a fall.

## Supplementary Information

Below is the link to the electronic supplementary material.

Supplementary Material 1

## Data Availability

Availability of data and materials. The datasets generated and/or analysed during the current study are not publicly available because the qualitative interview data contain potentially identifiable information and the participants did not consent to public data sharing.

## Abbreviations

ED: Emergency Department
PCC: Person – centred care
