# Supplementary material for: Sustaining agency after a fall: navigating healthcare and adjusting everyday life – a qualitative interview study
Source: BMC Geriatr. 2026 Oct 8;26:1373. doi: 10.1186/s12877-026-08418-2 (PMC13723096; doi:10.1186/s12877-026-08418-2)
Supplement: Supplementary file 1 [file 12877_2026_8418_MOESM1_ESM.docx]

**Interview Guide – Older Adults**

**Introduction**

I am a nurse studying older adults' experiences following a fall and treatment in the emergency department. I am interested in learning about your experiences following a fall and your contacts with healthcare services. I am interested in your own experiences and perspectives.

**Experiences of the fall**

- Can you tell me what happened when you experienced the fall?
- What were you doing that day?
- How do you remember the event?
- How did you experience the situation?
- How did you react at the time?
- What did you do afterwards?
- Do you think the fall could have been prevented in any way?

**Experiences of care and follow-up**

- How do you remember the care you received following the fall?
- What are your experiences of the care you received in different settings (e.g. at home, in primary care, in the ambulance service, in the emergency department, and during follow-up visits)?
- What aspects of the care worked well?
- Were there aspects of the care that could have been improved?
- Did you receive information about what would happen after your emergency department visit?

**Fall prevention and support**

- Have any measures been taken to prevent future falls?
- What is your opinion of the care you received following the fall?
- If you could choose, how would you have wanted the care to be organized?
- What type of support would you have wanted to help prevent another fall?

**Everyday life after the fall**

- Can you describe what your everyday life has been like since the fall?
- Has your everyday life changed since the fall?
- If yes, in what ways?
- How do you experience your everyday life today?
- How do you feel about these changes?

**Closing questions**

- Is there anything else you would like to tell me about your experiences?
- Is there anything we have not discussed that you think is important?
- Do you have any questions before we conclude the interview?

**Interview Guide – Relatives**

**Introduction**

I am a nurse studying older adults' experiences following a fall and treatment in the emergency department. I am interested in learning about your experiences related to your relative's fall and the care that followed.

**Experiences of the fall**

- Can you describe what happened when your relative experienced the fall?
- How were you involved at the time of the fall?
- What do you remember about the event?
- How did you experience the situation?
- How did you react at the time?
- Do you think the fall could have been prevented in any way?

**Experiences of care and follow-up**

- How do you remember the care your relative received following the fall?
- What are your experiences of the care your relative received in different settings (e.g. at home, in primary care, in the ambulance service, in the emergency department, and during follow-up visits)?
- What aspects of the care worked well?
- Were there aspects of the care that could have been improved?
- Did you receive sufficient information about what would happen after the emergency department visit?

**Fall prevention and support**

- Have any measures been taken to prevent future falls?
- What is your opinion of the care your relative received?
- If you could choose, how would you have wanted the care to be organized?
- What type of support would you have wanted your relative to receive to help prevent another fall?

**Everyday life after the fall**

- How has the fall affected your relative's everyday life?
- Has the fall affected your own everyday life?
- If yes, in what ways?
- How do you experience the situation today?
- How do you feel about it?

**Closing questions**

- Is there anything else you would like to tell me about your experiences?
- Is there anything we have not discussed that you think is important?
- Do you have any questions before we conclude the interview?
